# Application value of preoperative dual-source computed tomography in assessing the rupture site of thoracic aortic dissection

**DOI:** 10.1186/s13019-021-01729-y

**Published:** 2021-12-06

**Authors:** Fang Huang, Hong Wu, Qing-Quan Lai, Xiao-Ting Ke

**Affiliations:** Department of Radiology, The Second Affiliated Hospital, Fujian Medical University, Quanzhou, China

**Keywords:** Angiography, Aortic dissection, The rupture site

## Abstract

**Objective:**

To investigate the application value of dual-source computed tomography (DSCT) in preoperative assessment the rupture site of an thoracic aortic dissection (TAD).

**Methods:**

A retrospective analysis of preoperative DSCT, multislice computed tomography (MSCT), and transthoracic echocardiography (TTE) results of 150 patients with suspected TAD in our hospital was conducted, and the intraoperative findings or interventional treatment results were used as the diagnostic gold standard.

**Results:**

Of all 150 suspected TAD patients, 123 patients were confirmed to have TAD. The rupture site of TAD was in the ascending aorta in 46 patients, in the aortic arch in 13 patients, and in the descending aorta in 64 patients. The sensitivity of DSCT, MSCT, and TTE for locating the rupture site of the TAD was 100%, 93.5%, and 89.5%, respectively, and the specificity was 100%, 88.9%, and 81.5%. The differences were statistically significant. The distance between the actual rupture site and the one diagnosed by DSCT, MSCT, and TTE was 1.9 ± 1.2 mm, 5.1 ± 2.7 mm, and 7.8 ± 3.5 mm, respectively; the latter two were significantly worse than DSCT. The size of the rupture site diagnosed by DSCT, MSCT, and TTE was 1.5 ± 0.8 cm, 1.7 ± 0.9 cm, and 1.9 ± 1.0 cm, respectively. The size of the rupture site diagnosed by DSCT was not significantly different from the actual size of 1.4 ± 0.7 cm, while those by MSCT and TTE were.

**Conclusion:**

DSCT has high sensitivity and specificity in diagnosing the rupture site of TAD and can clearly locate the rupture site. It can be a preferred imaging method for TAD.

## Instruction

Thoracic aortic dissection (TAD) is an entity that seriously affects human health, with acute onset and extremely high mortality [1, 2]. Untreated TAD has a 24-h mortality rate of approximately 25% and a 2-week mortality rate of approximately 70% [2, 3]. Therefore, it is important to make a clear diagnosis and start corresponding treatments as soon as possible to reduce the mortality. Identifying the location and size of the rupture in thoracic aortic dissection plays an important role in the treatment, especially for Stanford B [4, 5]. Dual-source computed tomography (DSCT), multislice computed tomography (MSCT), and transthoracic echocardiography (TTE) are common examinations for suspected TAD patients. Each of the three methods has its own advantages and disadvantages [6–8]. Although TTE can be quickly examined and diagnosed at the bedside, it requires high operator’s experience and is limited to the diagnosis of descending aortic dissection due to pulmonary pneumatosis and other influences. MSCT is non-invasive, has short imaging time and high accuracy, and is relatively practical for TAD diagnosis. However, MSCT also has some limitations in cardiovascular examination, showing poor performance in dissection of aortic root and some small dissection. Compared with MSCT, DSCT is equipped with two sets of X-ray tubes and corresponding detectors. The resolution of DSCT is significantly higher than MSCT, and the location of TAD rupture is more accurate. Therefore, this was a retrospective study to analyze the diagnostic value of the three methods for the rupture site of TAD.

## Methods

The present study was approved by the ethics committee of our hospital. Family members and the patients had signed written informed consent for the examination.

### Research subjects

We retrospectively analyzed 150 suspected TAD patients treated in our hospital from January 2014 to October 2017. Inclusion criteria: suspected TAD patients by clinical diagnosis and/or those TAD patients with an imaging diagnosis in another hospital. Exclusion criteria: ① patients who were allergic to contrast agents; ② patients who had severe heart, liver, kidney, or other diseases and could not tolerate the imaging examinations; ③ patients whose imaging quality did not meet the diagnostic requirements. Of 150 patients, 120 patients were men and 30 patients were women. Their age was 49.2 ± 10.5 years. All the patients were identified by primary hospitals and then transferred to our hospital. Generally, for patients suspected of TAD, MSCT and TTE had been completed in primary hospitals, but the CT images could not be viewed in a timely manner. Therefore, DSCT was performed again to confirm the diagnosis in our center. The primary outcomes were the sensitivity and specificity of DSCT, MSCT, and TTE. The secondary outcomes were the distance between the actual rupture site and the one diagnosed by DSCT, MSCT, and TTE, and the size of the rupture site diagnosed by DSCT, MSCT, and TTE.

### Equipment and methods

The DSCT scanner was from Siemens (Germany) and could be used to perform both plain and enhanced scanning. The scan range was from the level of the first rib to the level of the pubic symphysis. The scanning parameters were as follows: rotation time 0.5 s, collimation width auto (64*0.625), pitch 0.65, reconstruction slice thickness 1.0 mm, tube A voltage 140 kV, tube B voltage 80 kV and auto mAs.

The MSCT scanner was a 64-row CT scanner from Philips (Netherlands) and was used for plain and enhanced scanning in this study. The scan range was from the level of the first rib to the level of the pubic symphysis. The scanning parameters were as follows: rotation time 0.5 s, collimation width auto (64*0.625), pitch 0.65, reconstruction slice thickness 1.0 mm, tube voltage 120 kV, and auto mAs.

In the enhanced scan of DSCT and MSCT, a high-pressure syringe was used to quickly inject contrast agent through the forearm vein, with injection rate of 4–5 mL/s and a total volume of 70 ml. When the concentration of contrast agent at the aortic root reached 100 Hounsfield units, the enhanced scanning was automatically performed. The original images were transmitted to the workstation for image postprocessing. The three-dimensional (3D) reconstruction technologies included multiplanar reconstruction (MPR) and curved planar reformation (CPR), maximum-intensity projection (MIP), shaded surface display (SSD), and volume rendering (VR).

TTE was performed using the GE-VV7 color Doppler ultrasound machine (USA) with the probe frequency of 2.5–5.0 MHz. Transthoracic and transsternal superior fossa views were obtained to observe the aortic root, ascending aorta, aortic arch, thoracic descending aorta, and abdominal descending aorta and their main branches. The lesion locations, intimal echo intensity of the dissected aorta, blood flow, and spectral characteristics in the true and false lumens of the aorta were documented in detail.

### Statistical analysis

The intraoperative findings or interventional treatment results were used as the diagnostic gold standard. Knowing the actual location of the rupture plays an important role in the treatment of TAD, especially the distance from the opening of the left subclavian artery. Therefore, the distance between the measured and actual rupture was compared in this paper. Missed diagnosis and misdiagnosis of the rupture site were defined as negative diagnosis of the rupture site. The letter “a” indicates that the DSCT, MSCT, TTE, and gold-standard results were all positive; “b” indicates that the DSCT, MSCT, and TTE results were positive and the gold standard was negative; “c” indicates that the DSCT, MSCT, and TTE results were negative and the gold standard was positive; and “d” indicates that the DSCT, MSCT, TTE, and gold-standard results were all negative.

Statistical analysis was performed using SPSS 19.0 software. According to the sensitivity of DSCT, MSCT, and TTE in the diagnosis of the TAD rupture site in the preliminary survey, the sample size of 150 was calculated in order to satisfy the following criteria: α = 0.05, two-tailed test, and power = 90%. The quantitative data are expressed as mean ± standard deviation and were compared with the independent-sample t test. The qualitative data were compared with the χ^2^ test. P < 0.05 was considered statistically significant.

## Results

Of all 150 suspected TAD patients, 123 patients were confirmed to have TAD. Among them, 59 cases were diagnosed as ATD of Stanford A (The rupture site of TAD was in the ascending aorta in 46 patients, in the aortic arch in 13 patients, and in the descending aorta in 64 patients), and 64 cases were diagnosed as Stanford B aortic dissection (The rupture site was in the descending aorta). All 150 patients underwent DSCT, MSCT, and TTE examinations, the primary results were shown in Table 1. Compared with the gold standard, the P values of the diagnoses by DSCT, MSCT, and TTE were all greater than 0.05, indicating that the accuracy of DSCT, MSCT, and TTE was not significantly different from that of the gold standard.

**Table 1:** The results of diagnosis TAD by DSCT, MSCT and TTE

Item	a	b	c	d	Sensitivity	Specificity
*DSCT*						
A	59	0	0	14	1	1
B	64	0	0	13	1	1
T	123	0	0	27	1	1
MSCT*						
A	56	2	3	11	0.949	0.846
B	59	1	5	13	0.922	0.929
T	115	3	8	24	0.935	0.889
*TTE*^*#*^						
A	54	2	5	10	0.915	0.833
B	56	3	8	13	0.875	0.800
T	110	5	13	22	0.895	0.815

T, total; A, type A thoracic aortic dissection; B, type B thoracic aortic dissection

For type A TAD, the sensitivity of DSCT, MSCT and TTE to the location of TAD rupture was 100%, 94.9% and 91.5%, respectively, while the specificity was 100%, 84.6% and 83.3%, respectively. For type B TAD, the sensitivity of DSCT, MSCT and TTE to the location of TAD rupture were 100%, 92.2% and 87.5%, respectively, and the specificity was 100%, 92.9% and 80.0%, respectively. Overall, the sensitivity of DSCT, MSCT and TTE to the location of TAD rupture was 100%, 93.5% and 89.5%, respectively. The sensitivity of DSCT was significantly higher than MSCT and TTE (P = 0.004 and P = 0.001, respectively). In specificity, DSCT, MSCT and TTE were 100%, 88.9% and 81.5%, respectively, for locating TAD rupture site. The specificity of DSCT was significantly higher than MSCT and TTE (P = 0.045 and P = 0.019, respectively). These results showed that the sensitivity and specificity of MSCT and TTE in the diagnosis of TAD were significantly lower than those of DSCT.

The secondary results of this study were described as follow. The distance between the actual rupture site and the one diagnosed by DSCT, MSCT, and TTE was 1.9 ± 1.2 mm, 5.1 ± 2.7 mm, and 7.8 ± 3.5 mm, respectively. The distance by the latter two methods was significantly different from that by DSCT (p = 0.021 and p = 0.008, respectively). The size of the rupture site diagnosed by DSCT, MSCT, and TTE was 1.5 ± 0.8 cm, 1.7 ± 0.9 cm, and 1.9 ± 1.0 cm, respectively. The size of the rupture site diagnosed by the DSCT was not significantly different from the actual size of 1.4 ± 0.7 cm (p = 0.668), while the size of the rupture site diagnosed by MSCT and TTE was (p = 0.041 and p = 0.023, respectively).

## Discussion

TAD is a critical condition. The differences in the location of the rupture site, the true and false lumens, and the affected vessels directly determine the treatments and the prognosis of the patient. Identifying the location and size of the rupture in thoracic aortic dissection plays an important role in the treatment, especially for Stanford B [4, 5]. The current preoperative imaging examinations include DSCT, MSCT, and TTE. Each of these three methods has its own advantages and disadvantages. This study analyzed and compared the results of these three methods through the diagnosis of rupture site of the TAD.

TTE is suitable for primary-care hospitals because it is a portable device that provides a low-cost and radiation-free examination. Many TAD patients are transferred from primary-care hospitals to higher-tier hospitals. Therefore, the value of echocardiography cannot be ignored, especially for patients who cannot be moved, in whom bedside ultrasound can be quickly performed for diagnosis [9, 10]. TTE can also be used to observe cardiac morphology, aortic regurgitation, cardiac function, and pericardial effusion [11, 12]. The sensitivity and specificity of TTE in the diagnosis of TAD in this study were 89.5% and 81.5%, respectively. However, TTE has some limitations. It requires a high-skilled sonographer and is interfered with by obesity, thoracic deformity, emphysema, and bronchitis. It has limited diagnostic value for aortic arch and descending aortic diseases [13].

MSCT has the advantages of short scan time and fast scan speed. It has become an important imaging method for clinical diagnosis of TAD. Recent studies have confirmed that MSCT improves the accuracy of rupture site detection from 50 to 90% [14, 15]. Due to the problem of resolution, MSCT has limitations in cardiovascular examination. Excessive heart rate, aortic valve regurgitation, coronary artery involvement, and motion artifacts can interfere with MSCT [16, 17]. In particular, the diagnosis of aortic root dissection is easier to be interfered with by these factors, which may cause MSCT to display poor-quality images [18]. The pulsation artifacts of the ascending aorta are mainly manifested as arc shadows within the lumen of the blood vessel, namely, “false endometrial slices” or “false lumen shadows” [19]. The TAD’s intima and true and false lumens often involve the ascending aorta, and its artifacts are similar to the signs of TAD. The rotation time of multilayer spiral CT tube is generally 0.5–1 s, which is similar to the time of the cardiac cycle of most patients. This affects the image quality to a certain extent and easily leads to misdiagnosis. Moreover, the torn intimal tissue is very thin and spiral-shaped. It can cause pulsation artifacts due to its swing in the cardiac cycle. The pulsation artifacts can easily affect the display of the TAD, especially if the rupture site is small. These may affect the diagnosis of a rupture site of the TAD.

DSCT scanners are based on ordinary 64-row CT and have two sets of X-ray tubes and two sets of detectors to acquire CT images. Because it uses two acquisition systems at the same time, the tube voltage and current of the two tubes can be adjusted separately, and the obtained data can be separately reconstructed to improve the temporal resolution [20]. DSCT greatly improves the temporal resolution and can complete a heart scan in 5–10 s. The image quality of cardiac images is no longer affected by the patient’s heart rate and arrhythmia, so reliable heart image acquisition is achieved. No beta-blockers are required for any heart rate condition. Noninvasive cardiac CT imaging can be used as a routine, like other CTs [21]. The total energy power of the two X-ray sources is 160 kW, which ensures excellent image quality even at the fastest scanning rate and table feed speed. DSCT has a 78-cm large-frame aperture and imaging field of view, a 200-cm scanning range, and the power of a high-voltage generator. It even can be used in tall and obese patients to perform a full-body scan without being limited by the patient’s size or physical condition. Moreover, this is can be done at very low radiation doses.

Here, the sensitivity and specificity of DSCT in the diagnosis of TAD were the same as those of the gold standard and were significantly higher than those of MSCT. Compared with MSCT, DSCT has the following advantages: 1. It is less affected by rapid heart rate. DSCT can image patients with tachycardia, premature beats, and arrhythmia and patients who can only hold their breath for a short time. 2. It can evaluate coronary arteries, valve reflux, and ventricular wall motion. 3. The effect of motion artifacts is smaller. 4. It provides dual-energy scanning to generate different information of the same anatomical structure in one scan. 5. The radiation dose is small and can be reduced to 50% of that of conventional CT in the heart CT scan.

Raptopoulos et al. suggested that the misdiagnosis rate of TAD with only one test method out of TTE, CT, and magnetic resonance imaging (MRI) was approximately 5% [22]. Therefore, in our center, we perform DSCT and TTE on all suspected TAD patients diagnosed in other hospitals to confirm the diagnosis and to fully evaluate the location and size of the rupture site, the involvement of the aortic valve, and the degree of pericardial effusion. In recent years, endovascular graft exclusion (EVGE) has gradually become one of the main methods for the treatment of type B TAD due to its simple and minimally invasive characteristics. The most important parameters to be evaluated in EVGE treatment are the accurate location of intimal rupture site and the identification of true lumen and false lumen. MSCT is easy to miss the number of cracks when the interlayer is small or the intima is twisted and torn seriously, while DSCT can clearly display the small cracks at the early stage of intramural hematoma and multiple small cracks at the twisted and torn intima. In this study, we found that DSCT could be better confirmed the location and size of the rupture site than MSCT and TTE.

This study was a retrospective case–control study and had the following limitations: 1. Most of the MSCT scans in this study were done in other hospitals, and biases in the diagnosis may have existed due to the individual experience and skills of the radiologists. 2. This was a single-center study with a small sample size. Larger, multicenter, randomized controlled clinical studies are needed to obtain more objective and valid results.

## Conclusion

DSCT is a non-invasive examination, and its accuracy for assessing TAD rupture sites is similar to that of the gold standard. Its diagnostic sensitivity and specificity are higher than those of MSCT and TTE. It can provide a more accurate diagnosis of the rupture site in TAD patients that MSCT and TTE. Therefore, we suggest that DSCT is more suitable as the preferred imaging method for TAD, and TTE is an important supplement for diagnosing aortic valve regurgitation, cardiac function, and pericardial effusion.

## Data Availability

Data sharing not applicable to this article as no data sets were generated or analyzed during the current study.

## Abbreviations

DSCT: Dual-source computed tomography
TAD: Thoracic aortic dissection
MSCT: Multislice computed tomography
TTE: Transthoracic echocardiography
